# Customized-individually-made origin® implants in total knee arthroplasty allow a reliable solution for accurate reproduction of planned implant positioning

**DOI:** 10.1186/s40634-023-00706-9

**Published:** 2023-11-28

**Authors:** Martin Sajan, Mohamad K. Moussa, Nicolas Lefèvre, Charles Payan, Eugénie Valentin, Alain Meyer, Yoann Bohu, Zeinab Khalaf, Olivier Grimaud, Antoinne Gerometta, Alexandre Hardy, Frédéric Khiami

**Affiliations:** 1 Hôpital de la Pitié Salpêtrière—AP-HP, 75013 Paris, France; 2 Clinique du sport, 36 Boulevard Saint-Marcel, 75005 Paris, France; 3 Hôpital Henri Mondor- AP-HP, 94000 Créteil, France

**Keywords:** Customized Individually Made TKR, Preoperative planning, Radiological measurements, Accuracy rate, Outliers, HKA alignment, Implant positioning

## Abstract

**Purpose:**

To evaluate the accuracy and reproducibility of a patient-specific, customized individually made (CIM) total knee replacement (TKR) using the ORIGIN® prosthesis.

**Methods:**

This was a prospective study conducted at a University Hospital from January 15, 2019, to April 30, 2021. The study included patients planned for an ORIGIN® CIM TKR procedure. Exclusion criteria included revision surgery, severe deformity, stiffness, or laxity. Evaluations were carried out using computed tomography scans performed 8 weeks preoperatively and 6 weeks postoperatively. The primary outcome measurements were the preoperative, planned, and postoperative CT scan alignment measurements including the Hip-Knee-Ankle (HKA) angle, mechanical Medial Distal Femoral articular surface Angle (mMDFA, distal alpha angle), Posterior Distal femoral articular surface angle (PDFA, posterior alpha angle), mechanical Medial Proximal Tibial articular surface Angle (mMPTA, beta angle) and posterior proximal tibial angle (PPTA). Secondary outcomes included the accuracy of implant positioning with percentage of outliers at 2° and 3°

**Results:**

The study encompassed 51 knees from 50 patients with mean age of 68.1 (SD = 8.89). The overall HKA angle deviated by -0.93° [95% CI: -1.45; -0.43], and the PDFA angle by -0.61° [95% CI: -1.07; -0.15], while the mMPTA exceeded planned values by 1.00° [95% CI: 0.57; 1.43]. The 3° outliers rate ranged from 3.9% for the mMPTA to 7.8% for the HKA alignment, with no outliers in mMDFA and PPTA. Similarly, the 2° outliers rate ranged from 15.7% for both the PDFA angle and mMPTA to 19.6% for the HKA alignment. The Bland–Altman plots further emphasized the precision of planned and post-operative angles across all measurements.

**Conclusion:**

The CIM TKR showed high accuracy and reproducibility, closely matching preoperative planning. The weakest accuracy at 3°-outliers is in the reproduction of the HKA alignment at 92.2% (range for all angle: 92.2–100%). Similarly, the weakest accuracy at 2°-outliers is in the reproduction of the HKA alignment at 80.4% (range for all angles: 80.4–92.2%).

**Supplementary Information:**

The online version contains supplementary material available at 10.1186/s40634-023-00706-9.

## Introduction

Despite all advances in total knee arthroplasty (TKA), a substantial percentage of patient’s dissatisfaction remain the main concerns in most performed arthroplasty, ranging from 5 to 16.3% [1, 2]. In the absence of technical errors and complication, dissatisfaction is often attributed to socio-economic-demographic factors, psychological factors, alignment philosophy chosen, and prosthesis design [1, 3–7].

Anatomical studies have shown considerable variations of knee anatomy according to gender and ethnicity [3, 8]. This exposes to a substantial risk of femoral overhang and alignment issues when using over the shelf non-customized prosthesis [3, 9–11]. A study by Mahoney et al. highlights that femoral overhang exceeding three millimeters was present in 40% of men and 68% of women [9], and was correlated with a 2-odd increase in anterior knee pain at 2 years post-operatively [9].

s.

In an effort to theoretically reduce the dissatisfaction rate, the kinematic alignment which use measured bone resection to restore patient’s prearthritic knee anatomy, was introduced [6, 12, 13]. Rivière et al. found in their systematic review, that this technique can provide faster recovery and generate better functional outcomes, than mechanically aligned TKA, especially for osteoarthritic patients with slight to mid constitutional knee frontal deformity [6]. In patients with significant deformity, the restricted kinematic alignment technique, offer a more adapted option to decrease early failure, by providing a comprehensive algorithm for corrections of Hip-Knee-Ankle (HKA) axis and other mechanical angles [3].

In recent years, the concept of customized individually made (CIM) anatomical prostheses has emerged [5, 14, 15] to address accuracy problems in TKR procedures. Although their potential clinical benefits is not well established in literature [11, 16], recent investigation of the new ORIGIN® CIM prosthesis showed significant improvement in various clinical and patient-reported outcomes including the Knee Society Score (KSS), range of motion, and the Forgotten Joint Score (FJS) at 1-year follow-up [17]. These individualized patient’s specific implants, created from cross-sectional imaging of the lower limb, are designed with the triple target to respect the patient's anatomy, minimize bone cuts, and restore the most physiological joint kinematics possible [5, 7].The incorporation of machine learning in the automatic customization process has further contributed to the accuracy and success of CIM TKR [18], where these prosthesis are now available for primary as well as for revision hinged arthroplasty [19–21].

The aim of this study was to assess the positioning accuracy of CIM prostheses as measured by the reproducibility of the planned mechanical angles, with post-operative mechanical angles assessed using computed tomography (CT) scan.

## Material and methods

### Study design

This was a prospective, mono-centric observational study targeting consecutive patients operated with CIM TKR, specifically with ORIGIN® total knee prostheses (Symbios, Yverdon-les-Bains, Switzerland), between January 15, 2019, and April 30, 2021, at a University Hospital in Paris.

Institutional review board approval was obtained (Number 20220823170901).

### Inclusion and exclusion

All patients planned for ORIGIN® CIM primary TKR were included in the study. Exclusion criteria included patients with previous knee surgery, severe deformity (> 15° in the coronal plane), severe stiffness (extension deficit > 15°, flexion range < 90°), severe laxity (> 10° in varus, > 15° in valgus) or bone loss, and patients unable to comply with the study protocol and follow-up requirements.

### Outcome measures

The primary outcome measurements were the preoperative, planned and postoperative alignment CT scan measurements, such as the HKA angle, mechanical Medial Distal Femoral articular surface Angle (mMDFA, distal alpha angle), Posterior Distal femoral articular surface angle (PDFA, posterior alpha angle), mechanical Medial Proximal Tibial articular surface Angle (mMPTA, Beta angle) and posterior proximal tibial angle (PPTA). This also includes the percentage of patients where all measurements were within the safe zone at each stage of the procedure.

The secondary outcome measurements included the accuracy of implant positioning assessed by the percentage of cases within outliers of 2° and 3° and respective accuracy rate at these thresholds. This corresponds to the most commonly used definition of outliers in the literature, which defines them as deviations of more than 3° from the target value [22, 23]. An outlier of 2° was defined for patients exhibiting a deviation > 2° postoperatively compared to the planned measurement. This criterion is used in studies seeking a more detailed analysis of the precision [24].

### Prosthesis customization and 3D planning methodology

The utilized CIM ORIGIN® prosthesis was an anatomic posterior-stabilized prosthesis created from cross-sectional CT imaging of the lower limb (hip, knee, ankle), performed 8 weeks prior to surgery, following a standardized scanner protocol provided by the manufacturer [25], and a 64-slice multidetector scanner (Siemens® Sensation, Munich, Germany). The reconstruction and planning were performed using the KNEE PLAN® software (Symbios, Yverdon-les-Bains, Switzerland) (Appendices 1 and 2). All these measures were done by a qualified referring engineer from the manufacturer and validated by the treating surgeon case by case.

The alignment principles adopted were those of restricted kinematic alignment that aim to closely approximate the kinematics of the native knee and maintains the patient's preoperative deformity within a safe zone, while minimizing the risk of oversizing, improving bone coverage, avoiding bone overhangs, and reducing the thickness of bone cuts [3, 26]. The safe zone was defined in compliance with restricted kinematic alignment principle [3], as a deformity in the frontal plane (HKA) ≤ 3° and mMDFA or mMPTA ≤ 5° of obliquity relative to the mechanical axis[3]. PPTA was limited to a maximum of 5° [3].

**Fig. 1:**
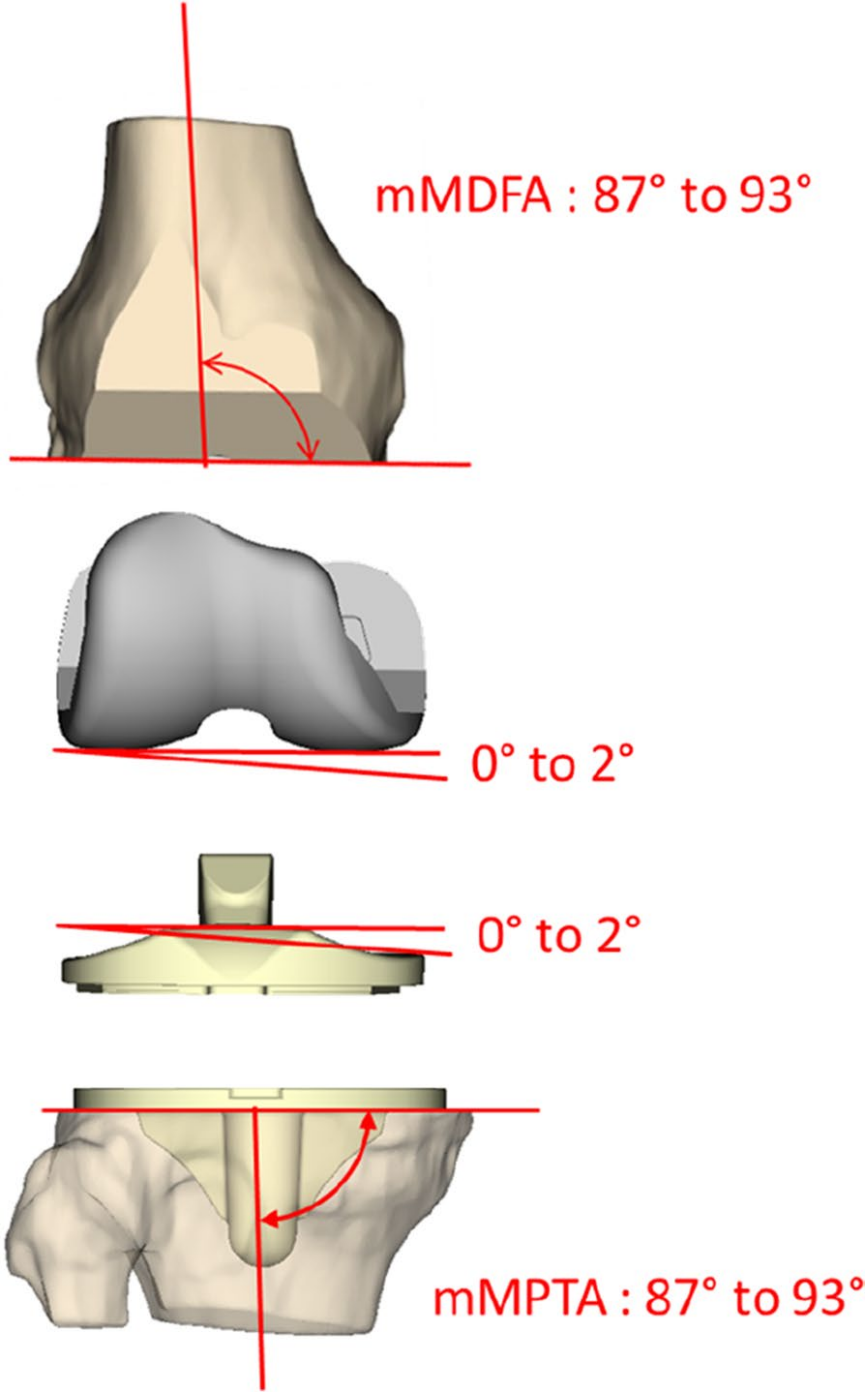
Illustration of the joint line obliquity correction with ORIGIN® implant (Symbios, Yverdon-les-Bains, Switzerland)

Patients outside this range were brought back into this zone by adjusting the bone cuts primarily on the tibial side. The reproduction of articular obliquity was distributed between the offset of the implants and the bone cuts (Fig. 1). Up to 3° of obliquity could be considered in the bone cuts, while up to 2° could be considered in the offset of the insert or the condyles.

### Surgical technique

Midvastus approach was used for all intervention and without the use of a pneumatic tourniquet. Patellar resurfacing was performed systematically.

### Post-operative scanner analysis

In the postoperative period, all patients underwent a second low-dose control CT scan at 6 weeks.

**Fig. 2:**
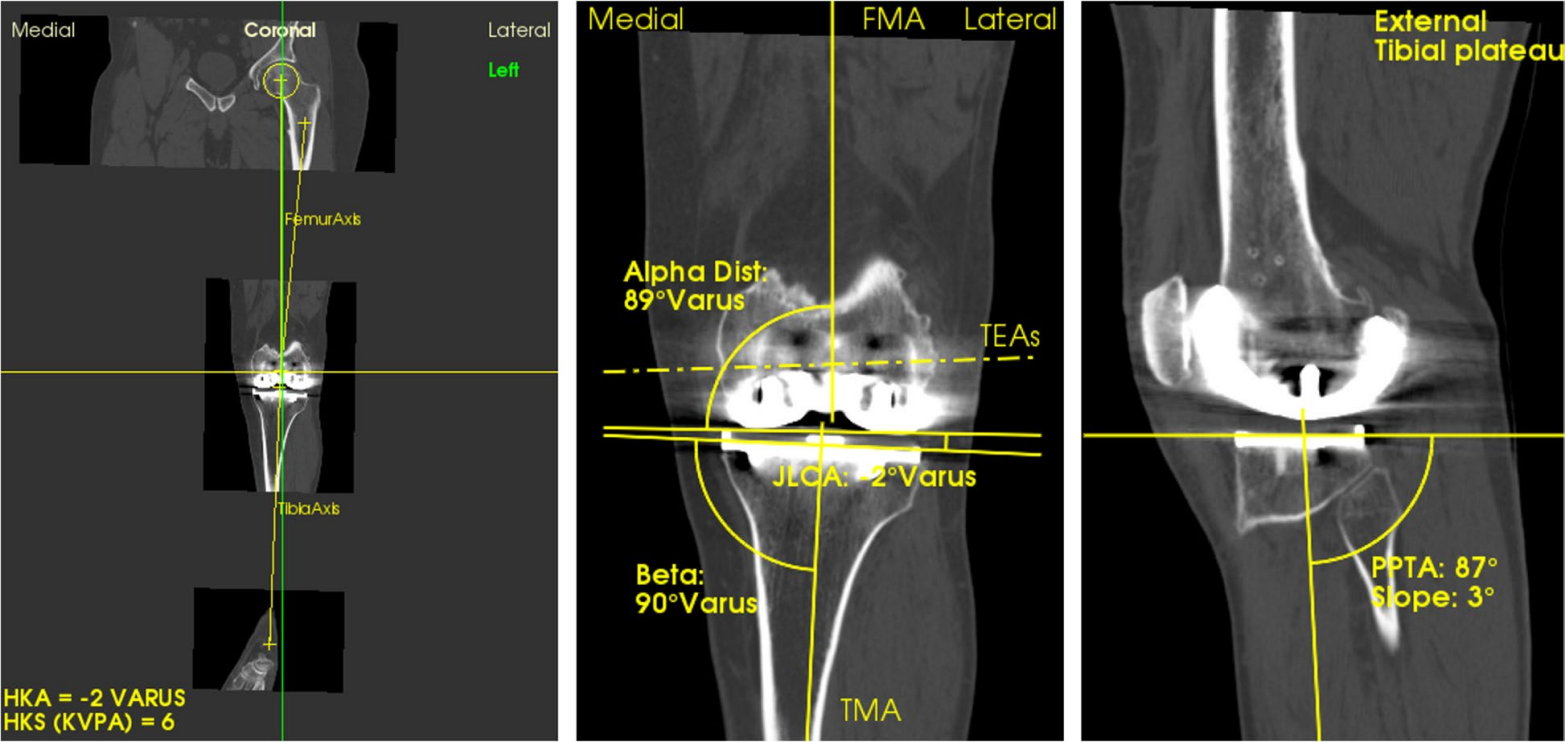
Postoperative analysis of Hip Knee Ankle (HKA) angle, tibial slope, distal alpha, beta angle, and posterior proximal tibial slope (PPTA)

**Fig. 3:**
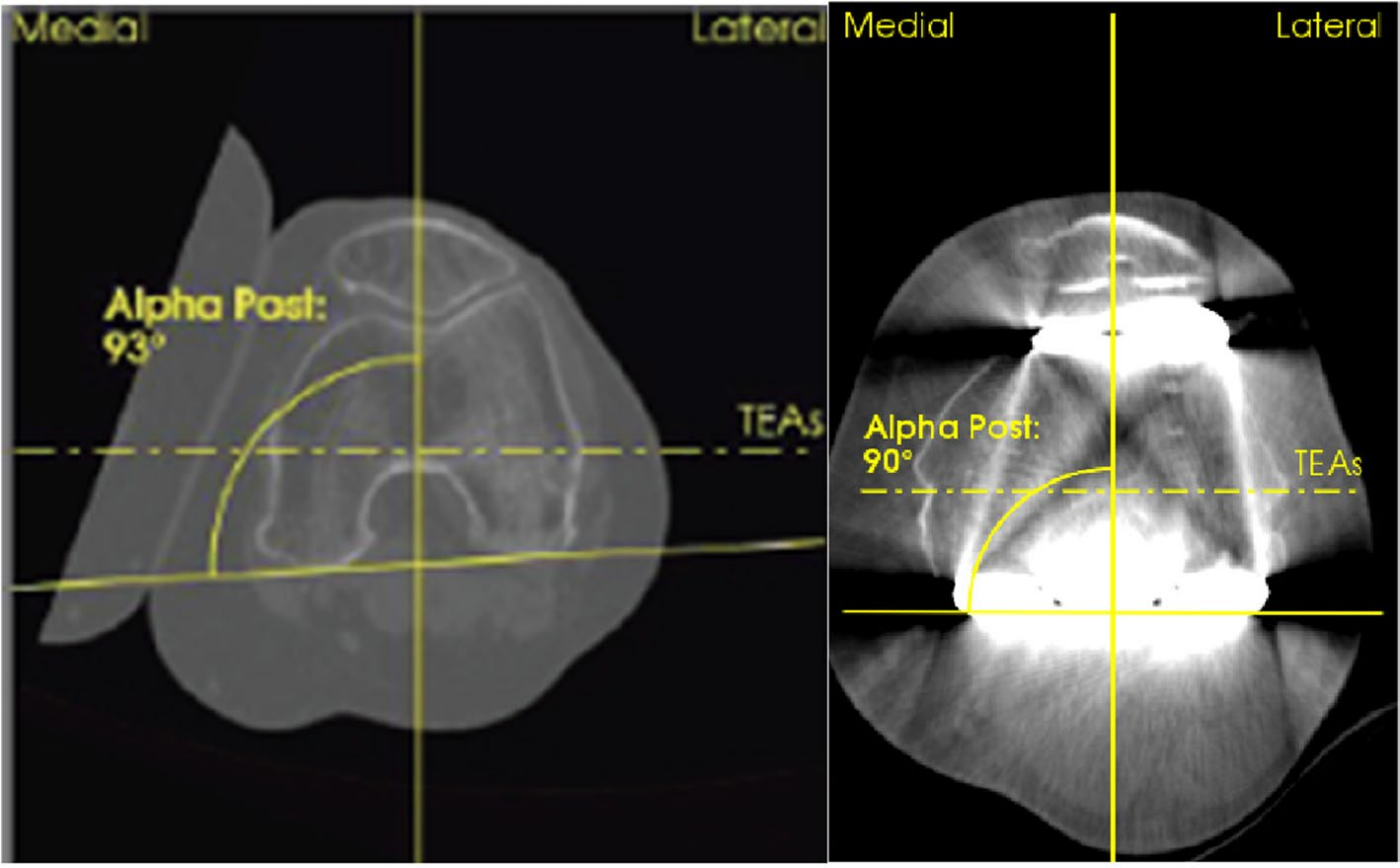
Preoperative (left) and postoperative (right) analysis of the posterior alpha angle

The radiological analysis of these CT scans was conducted with the help of the KNEE PLAN® software (Symbios, Yverdon-les-Bains, Switzerland) and the analysis of the same qualified referring engineer who performed the preoperative measures (Fig. 2, 3).

### Study population

Fifty-four patients (55 knees) met the inclusion criteria. Of these, three patients were lost to follow-up, and one patient died, leaving an analysis population of fifty patients (51 knees).

### Statistical analysis

All results were analyzed using Microsoft Office Excel™ (Redmond, USA) and R Studio™ (Vienna, Austria) software. Variables were expressed qualitatively or quantitatively depending on their nature.

The paired samples t-test or the Wilcoxon signed-rank test was utilized for the comparison of means and the McNemar test was conducted to evaluate the agreement between planned and post-operative proportions.

The Bland–Altman plot was used to visually assess the agreement between the two measurements. The dispersion of data points around the mean difference line provided insights into the level of agreement between planned and post-operative measurements.

The *p*-value was considered significant if it was less than 0.05.

## Results

### Demographic characteristics

The mean age at surgery was 68.1 (SD = 8.89), with a male prevalence of 64.7% (33 males) and 35.3% females (18 females). The distribution of surgeries involved more right knees (52.9%) than left (47.1%). The average BMI was 28.1 (SD = 3.96), with 31.4% of patients classified as obese (BMI > 30).

### Changes in pre-operative, planned, and post-operative radiological measurements (Table 1)

**Table 1:** Changes in pre-operative, planned, and post-operative alignment measurements

	**Pre-Operative Measures**	**Planned versus post-operative measures**			
		Planned Measures	Post-Operative Measures	Difference between Planned and Post-Operative Measures [95% CI]	*P*-value
**Overall HKA Population (°):** mean (range)	177 (162–188)	179.5 (176–182)	178.5 (171–183)	-0.93 [-1.45; -0.43]	0.001
**Distal Alpha angle (mMDFA) (°): mean (range)**	92.8 (86–99)	91.6 (88–94)	91.5 (86–96)	-0.15 [-0.52; 0.21]	0.4
**Posterior Alpha angle (PDFA) (°): mean (range)**	92.5 (89–97)	91.4 (86–96)	90.7 (86–95)	-0.61 [-1.07; -0.15]	0.01
**Beta angle (mMPTA) (°): mean (range)**	87 (82–93)	87.8 (86–90)	88.7 (86–92)	1.00 [0.57; 1.43]	0.001
**PPTA (°): mean (range)**	85.8 (85–88)	85.9 (85–88)	86.2 (82–89)	0.28 [-0.06; 0.62]	0.1

The transition from pre-operative to post-operative measures shows a general trend toward alignment with planned measures.

Comparing the planned measures with the post-operative outcomes, results were comparable with slight deviations observed in some of the angles not exceeding an average of 1°. Specifically, the overall HKA angle showed a minor deviation of -0.93° [95% CI: -1.45; -0.43] (*p* = 0.001), and the PDFA angle exhibited a reduction of -0.61° [95% CI: -1.07; -0.15] (*p* = 0.01). However, the Beta angle (mMPTA) exceeded the planned average by 1.00° [95% CI: 0.57; 1.43] (*p* = 0.001). The differences in Distal Alpha angle (mMDFA) and PPTA were not statistically significant.

### Accuracy of implant positioning and outliers (Table 2)

**Table 2:** Accuracy of implant positioning, assessed by the percentage of cases falling within 2° and 3° outliers, as well as the respective accuracy rates at these thresholds

**Alignment measurement**	**Three-degree threshold assessment**		**Two-degree threshold assessmen**t	
	Outliers 3° (total = 51)	Accuracy at 3°	Outliers à 2° (Total = 51)	Accuracy at 2°
**HKA**: n (%)	4 (7.8%)	47/51 (92.2%)	10 (19.6%)	41/51 (80.4%)
**Distal Alpha angle (mMDFA):** n (%)	0 (0%)	51/51 (100%)	0 (0%)	51/51 (100%)
**Posterior Alpha angle (PDFA):** n (%)	3 (5.9%)	48/51 (94.1%)	8 (15.7%)	43/51 (84.3%)
**Beta angle (mMPTA):** n (%)	2 (3.9%)	49/51 (96.1%)	8 (15.7%)	43/51 (84.3%)
**PPTA:** n (%)	0 (0%)	51/51 (100%)	4 (7.8%)	47/51 (92.2%)

For the HKA alignment, 4 cases (7.8%) were 3° outliers, resulting in an accuracy rate of 92.2% (47 out of 51). When assessed at the 2° threshold, 10 cases (19.6%) were outliers, yielding an accuracy rate of 80.4% (41 out of 51).

Regarding the distal alpha (mMDFA) angle, there were no outliers at either the 3° or 2° thresholds, hence a 100% accuracy rate was recorded in both cases. For the posterior alpha (PDFA) angle, 3 cases (5.9%) were 3° outliers, resulting in an accuracy rate of 94.1% (48 out of 51), and there were 8 outliers (15.7%) at 2°, leading to an accuracy rate of 84.3% (43 out of 51).

In terms of the beta (mMPTA) angle, there were 2 cases (3.9%) at 3°, producing an accuracy rate of 96.1% (49 out of 51), and 8 cases (15.7%) were outliers at 2°, yielding an accuracy rate of 84.3% (43 out of 51). Lastly, for the posterior tibial slope (PPTA), there were no outliers at the 3° threshold, corresponding to a 100% accuracy rate, whereas 4 cases (7.8%) were 2° outliers, leading to an accuracy rate of 92.2% (47 out of 51).

**Fig. 4:**
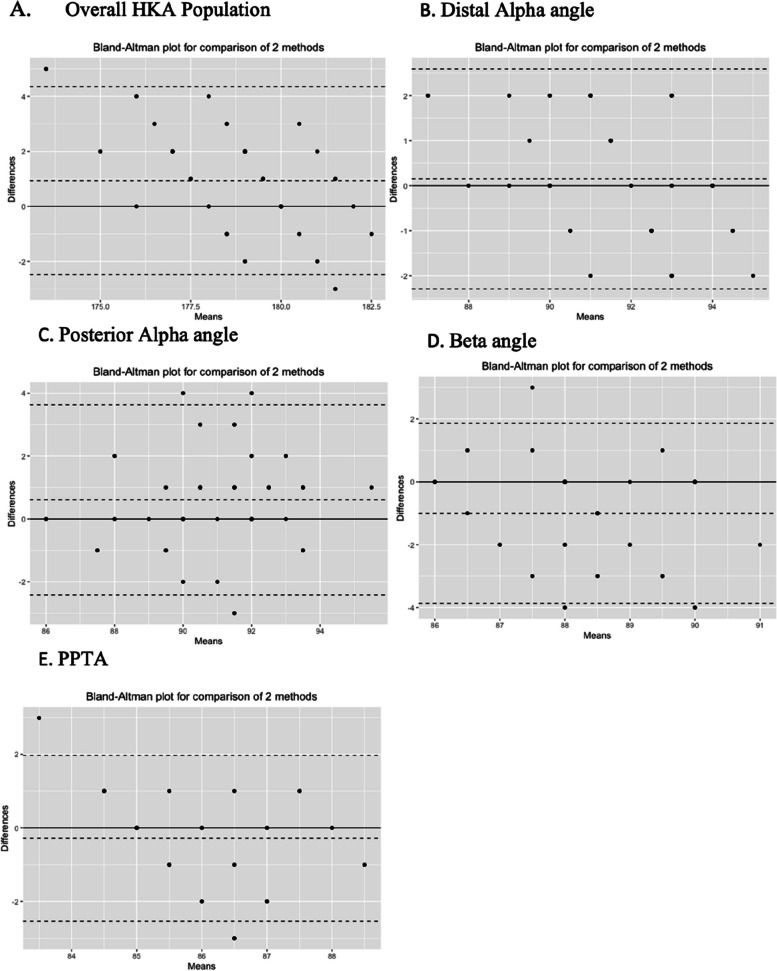
The Bland-Altman plots showing a consistent agreement between planned and post-operative angles across all measurements

The Bland–Altman plots supported the accuracy rates observed, revealing a consistent agreement between planned and post-operative angles across all measurements (Fig. 4).

## Discussion

The main finding of our study is that Origin® CIM TKR reliably reproduce the intended alignment plans in the post-operative phase with a negligible mean difference between the planned and post-operative measures across all categories, the largest being a mere 1° in HKA.

These data demonstrate that CIM TKR, offers high accuracy rates for implant positioning for all angles measured, with relatively low outlier percentages. The accuracy rates were above 80% in all cases, and for some measurements like mMDFA and PPTA at the 3° threshold, the accuracy was 100%. The Origin® prosthesis has been on the market since 2018, and there are currently very few studies on this implant [19]. Only one study discusses the placement accuracy of the ORIGIN® prosthesis, through postoperative radiographic analysis of 258 prostheses [19]. It found an accuracy rate of 84% with CIM ORIGIN® prosthesis [19].

### Global knee alignment: HKA

Our results demonstrated a 19.6% and 7.8% rate of outliers outside the 2° and 3° target zones, respectively. Our results remain better than those found in the literature using conventional instruments [27, 28], and are close to the best results found with custom cutting guides [29]. Levengood et al. demonstrated a lower rate of 2° outliers, at 15.8% [30]. Wunderlich et al. conducted a retrospective analysis to investigate the reconstruction of the HKA angle in CIM TKA implant system (iTotal® CR G2, ConforMIS) compared to an off-the-shelf knee replacement system (Vanguard®^s^ CR, Zimmer Biomet) [31]. A total of 562 TKA patients (283 CIM TKA, 279 off-the-shelf TKA) were included in the analysis. They found comparable corrected postoperative HKA in the CIM group was 179.0° ± 2.8°, and 179.2° ± 3.1° in the control group (p = 0.34). The rate of outliers outside of the ± 3° target zone, was similar in both groups (32.9%) [31].

Although Bonnin et al. based their study on a restricted kinematic alignment strategy, the authors' postoperative alignment goal differs from our study, with a target HKA range between 175 and 183°, which is larger than ours. Furthermore, postoperative measurements are taken on radiographs rather than CT scans. The authors report a significant difference of 1.3° in HKA between preoperative radiological and preoperative CT measurements [19].

In contrast, Kumar et al. found a difference in favor of conventional TKR compared to CIM TKR using iTotal® implant. They reported a higher rate of coronal malalignment > 3° (26.6%) compared to conventional technique (9.9%) (*p* < 0.01). This study, however, reported technical errors in the CIM TKR group, which may have influenced these results [32].

When it comes to navigated TKR, Hetaimish et al. performed a meta-analysis including 2541 patients and comparing postoperative alignment results with standard instrumentation and navigation, and found an HKA outlier rate of 30.1% with conventional instrumentation and 12.8% with navigation, with these figures rising to 40.4% and 20% for 2° outliers [24]. Our HKA outlier numbers are therefore lower than the navigation numbers in this study and approach the 4% observed by Todesca et al. [28]. Furthermore, a recent study by Klasan et al. compared robotic-assisted surgery with computer-assisted surgery in TKA, and found no significant differences in alignment outcomes or outlier reduction [33]. The findings from our study highlight the potential of CIM TKA as a viable alternative to both navigation and robotic-assisted techniques. The low rates of HKA alignment outliers are especially noteworthy when contrasted with the rates reported for navigated and robotic-assisted surgeries.

Since the accuracy of CIM TKR implant is primarily influenced by the application of CIM cutting guides, it is reasonable to compare our results with those of standard prostheses placed with CIM cutting guides. Sariali et al. find an outlier rate of 3° on HKA, 10% higher than ours [27]. This result is particularly interesting as he uses the FIRST® cutting guides (Symbios, Yverdon-les-Bains, Switzerland) which are those used with our prosthesis, and like us, their results are analyzed with a postoperative scanner.

### Femoral component alignment (distal and posterior Alfa angle):

Our data demonstrates perfect accuracy in the distal femoral cuts (mMDFA) with zero outliers at 2° and 3°, which aligns with the negligible mean difference between our planned and postoperative measurements. These findings are consistent with those reported by Bonnin et al., who found a comparable difference of -0.5° with the same prosthesis [19].

The PDFA is a crucial indicator of component rotation, and failing to replicate accurate rotation could ultimately impact patient satisfaction rates [34]. In our study, we observed a slight increase in outliers, with 5.9% at 3° and 15.7% at 2°. Nevertheless, the mean discrepancy between our planned and postoperative measurements is minimal, ranging from 0.1° to 0.7°, compared to the 0.5° noted in Bonnin et al.'s study [19]. Our accuracy results remain better than the results of Hetaimish et al., who documented a less satisfactory precision with 18.8% of outliers using navigation and 14.5% with mechanical ancillaries [24]*.* Sariali et al. reported comparable results to ours, with a 3° outlier rate of 5% when planning a 90° angle measured between the femoral axis and the bi-condylar axis [27]. This was significantly better than the 20% they found using mechanical technique [27]. These results confirm those found by Mannan et al. who reported a statistically significant improvement in the precision of the posterior alpha from the cutting guides compared to conventional ancillaries [34].

### Tibial component alignment (Beta angle and PPTA):

On the tibial side, our data align with existing literature, where Bonnin et al. who found 0.5° difference between planning and post-operative measurements (compared to -0.9) in our study [19].In their randomized prospective study comparing outlier rates for the beta angle between navigation, custom cutting guides, and conventional intra-medullary and extra-medullary tools, Zahn et al. identified superior statistical precision with navigation and intra-medullary guides [35]. However, they found a 3° outlier rate of 4% for navigation, 6.7% for intra-medullary, 13.3% for extra-medullary, and 18.7% for cutting guides [35]. Our results align closely with their excellent findings for navigation. Studies investigating CIM cutting guide shows slightly lower rate of 3° outliers: with Gaukel et al. reporting 2.3% [29] while Sariali et al. reported a perfect rate of 0% [27].

Regarding the assessment of the tibial slope (PPTA), we report very promising results, with no outliers at 3° and only 7.8% outliers at 2°. The observed difference between the planned slope and the postoperative slope is comparable to the -0.4% reported by Sariali et al. [27]. Similarly, none of the patients showed a reversed slope, and only 5 patients (9.8%) presented a slope > 5°, outside the established objectives with restricted kinematic alignment. The tibial slope is described by many authors as the most challenging element to manage with various tools. While navigation has proven its accuracy [22], Todesca et al. found 11% outliers at 3° with navigation and 13% with conventional tools [28].

### Clinical outcomes

CIM TKR represents an evolving technology that aims to enhance patient outcomes through improved accuracy. Investigations like ours adds data to the existing literature, providing the evidence needed to establish the effectiveness of CIM TKR. Victor et al.’s review found out 17 studies on CIM TKR, with 15 of them focusing on the iTotal custom-made TKA (Conformis, Massachusetts, USA), one on the Origin implant (Symbios, Yverdon-Les-Bains, Switzerland), and one not documenting the implant type [21]. Some studies demonstrated improved clinical outcomes of CIM TKA compared to traditional implants, including lower transfusion rates, lower adverse event rates, superior mechanical axis reproduction, and more consistent coronal alignment of the femoral component. However, these benefits cannot be safely retained because most studies were limited by biases such as absence of comparative groups, and study financed for commercial aim and presence of conflict of interest.

### Limitation

The primary limitation of our study is the lack of a control group and the inclusion of a small number of patients. Another limitation of our study is the use of a CT scan to carry out our post-operative measurements. In fact, most literature utilizes weight-bearing radiographs. In their work, Bonnin et al. found a significant difference of 1.3° between radiological and CT pre-operative measurements, which shows the limitations of comparing our results with those of studies that used post-operative radiographs. However, this also confirms one of the strengths of our study, which is the use of the same measurement instrument pre- and post-operatively by the same qualified engineer. This enhances the validity of our results regarding accuracy, particularly on outliers.

## Conclusion

This study indicates that ORIGIN® implants in CIM TKR provide satisfactory and often superior positioning accuracy in TKA relative to existing literature, with the lowest accuracy for HKA alignment reproduction noted at 92.2% for 3°-outliers and 80.4% for 2°-outliers.

### Supplementary Information

**Additional file 1: Appendix 1. **Preoperative analysis Knee Plan® (Symbios, Yverdon-les-Bains, Switzerland).** Annexe 2. **Preoperative planning using the Knee Plan^®^ (Symbios, Yverdon-les-bains, Suisses)

## Data Availability

Data are available upon reasonable request.
