# Supplementary material for: Customized-individually-made origin® implants in total knee arthroplasty allow a reliable solution for accurate reproduction of planned implant positioning
Source: J Exp Orthop. 2023 Nov 28;10:123. doi: 10.1186/s40634-023-00706-9 (PMC10684845; doi:10.1186/s40634-023-00706-9)
Supplement: Supplementary file 1 — Additional file 1: Appendix 1. Preoperative analysis Knee Plan® (Symbios, Yverdon-les-Bains, Switzerland). Annexe 2. Preoperative planning using the Knee Plan® (Symbios, Yverdon-les-bains, Suisses) [file 40634_2023_706_MOESM1_ESM.docx]

Annexe


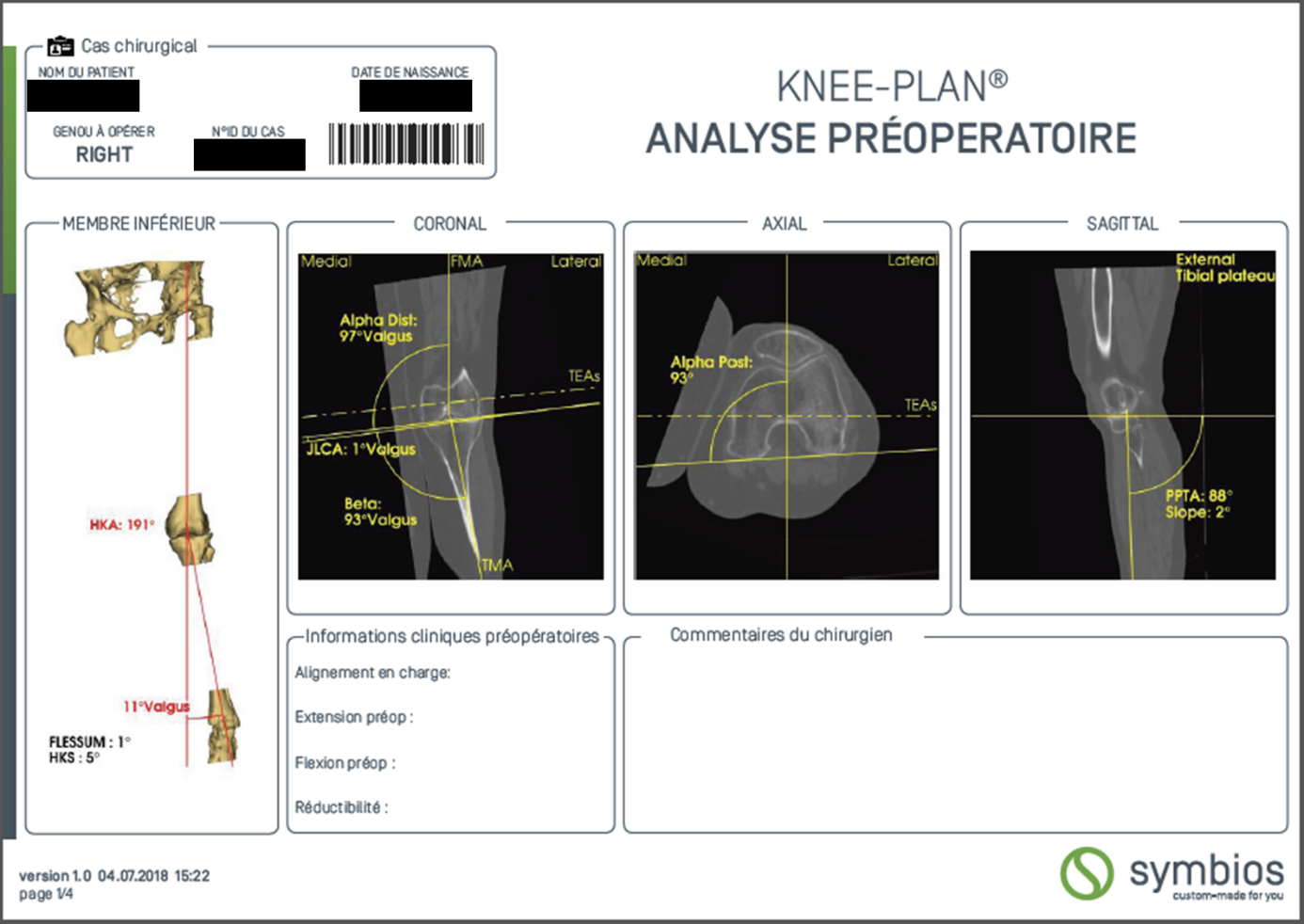


Appendix 1: Preoperative analysis Knee Plan® (Symbios, Yverdon-les-Bains, Switzerland)

TEAs = Trans-epicondylar Axis; Preoperative posterior alpha angle = angle measured between the femoral mechanical axis and the posterior condylar axis; JLCA (Joint Line Convergence Angle) = angle of convergence between the tibial and femoral articular lines; Distal alpha angle = angle measured between the distal femoral articular line and the femoral mechanical axis; Preoperative beta angle = angle measured between the proximal tibial articular line and the tibial mechanical axis; HKA = Hip-Knee-Ankle angle; HKS = Hip-Knee-Shaft angle; PPTA (Posterior Proximal Tibial Angle) = posterior tibial slope.


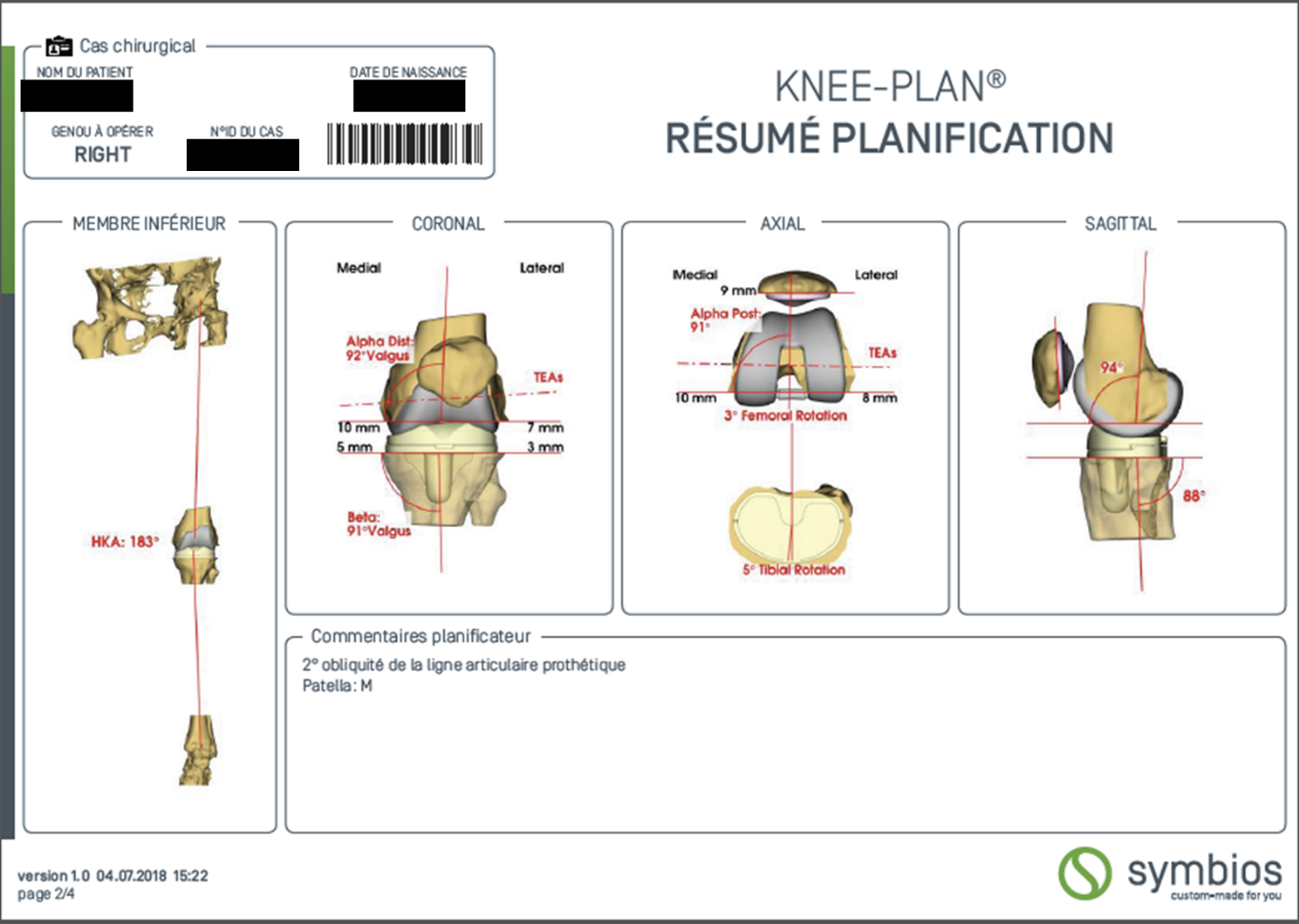


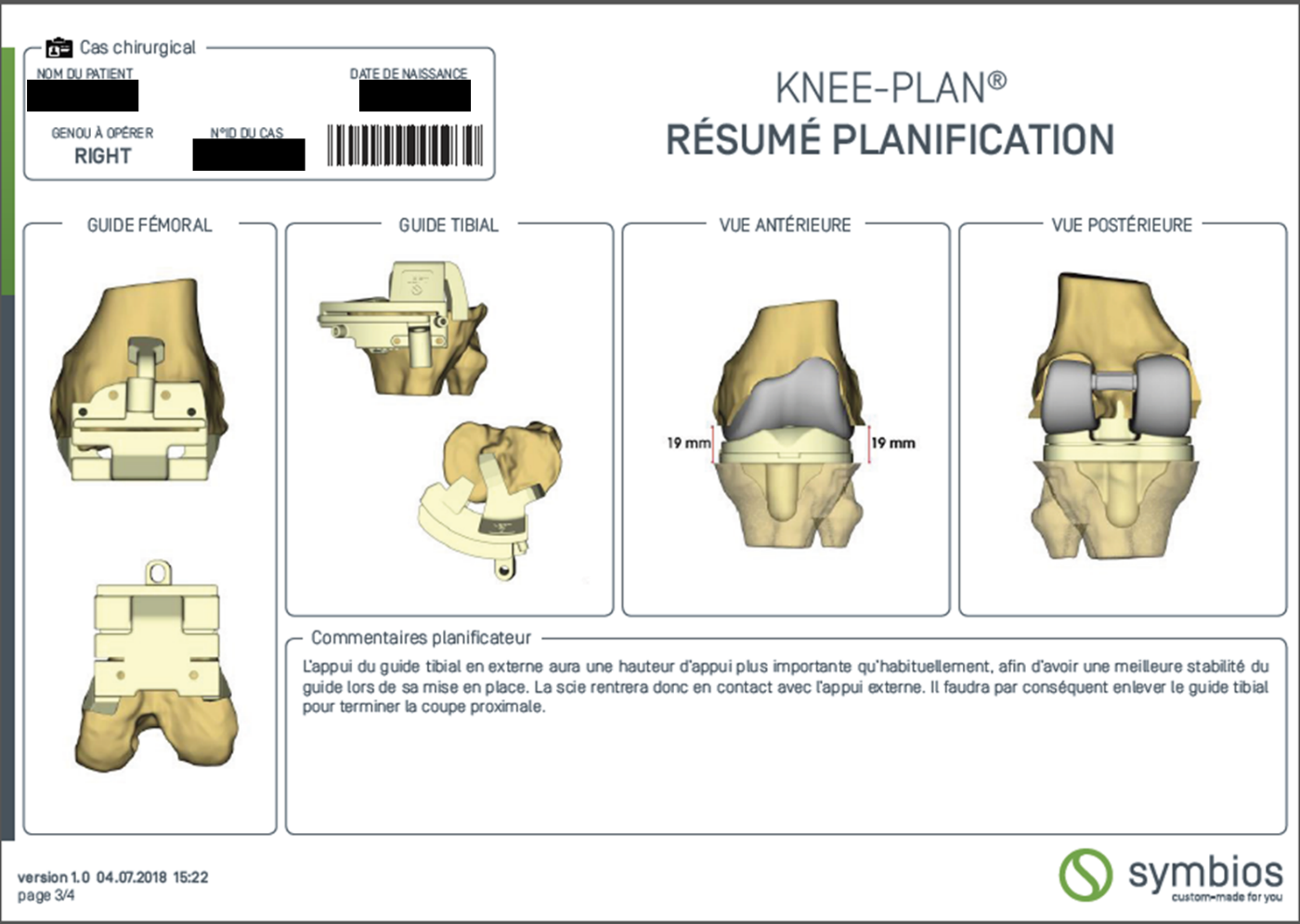


Annexe 2 : preoperative planning using the Knee Plan^®^ (Symbios, Yverdon-les-bains, Suisses)
